# Cetaceans along the southeastern Brazilian coast: occurrence, distribution and niche inference at local scale

**DOI:** 10.7717/peerj.10000

**Published:** 2020-10-05

**Authors:** Giovanna Corrêa e Figueiredo, Karina Bohrer do Amaral, Marcos César de Oliveira Santos

**Affiliations:** 1 Department of Biological Oceanography, Universidade de São Paulo, São Paulo, São Paulo, Brazil; 2 Department of Zoology, Universidade Federal do Rio Grande do Sul, Porto Alegre, Rio Grande do Sul, Brazil

**Keywords:** Niche overlap, Ecological niche modeling, *Balaenoptera edeni*, *Stenella frontalis*, *Tursiops truncatus*, *Steno bredanensis*, *Sotalia guianensis*, *Pontoporia blainvillei*

## Abstract

It is deemed important to understand cetacean occurrence and distribution to comprehend their ecological roles. The geographical occurrence of species’ niche can be used to better describe their potential distribution. The niche can be defined using environmental variables. Those variables are considered static and not affected by biological activities. The present study goal was to assess the occurrence and distribution of cetaceans along the southeastern Brazilian coast, as well as to define the fundamental and realized niche of each species and to investigate niche overlap at local scale. The environmental requirements for each species were also investigated throughout statistical tests. Sighting data were obtained through oceanographic surveys conducted between 2012 and 2015. The environmental variables available on MARSPEC and the software NicheA were used for the ecological niche modeling. A total of twelve cetacean species were identified and the potential distribution areas of the six commonest ones were defined. Even though the species presented different environmental preferences, most of them had partial overlap among niches and potential distribution areas. The environmental heterogeneity of the study area might support the co-occurrence of different species with different environmental requirements.

## Introduction

Cetaceans are inserted in Cetartiodactyla order (sensu (Gatesy, 2009). Currently they are composed by 14 species from the suborder Mysticeti and 75 species from the suborder Odontoceti (Committee On Taxonomy, 2019). They are fully adapted to aquatic life and can exhibit different adaptations according to the environment they occupy. Cetaceans can be found from estuarine shallow waters to bathypelagic zones, where they can reach up to 3,000 m of depth (e.g., Berta, Sumich & Kovacs, 2006; Ballance, 2009; Chivers, 2009; Schorr et al., 2014). They are also present in waters from −2 °C, in polar regions, to more than 30 °C in coastal and tropical areas (Ballance, 2009). The presence of cetaceans in various environments and their distribution do not occur randomly, but can be directly or indirectly related to environmental variables, like salinity and temperature, to the oceanographic regime and to the distribution of prey (Gregr et al., 2013). Besides, different populations from the same species are known to present flexible behavioral traits and distinct strategies in the way they use the area when occupying distinct geographical areas (Bannister, 2009).

Cetaceans have been reported in the Southeastern Brazilian Continental Shelf (SBCS) since the whaling period (Ellis, 1969). Following records relied on single data from stranded animals (e.g., Sawaya, 1938; Carvalho, 1938; Carvalho, 1969), and then on occasional sightings reported in non-scientific medias (e.g., Santos et al., 2010). Even though extremely important, stranding data show a high probability of bias based on occasional transport of carcass by oceanographical currents (Geraci & Lounsbury, 1993; Williams et al., 2011). Only in the early 2000s, systematic surveys were conducted in order to specifically describe cetacean occurrence and distribution along the Brazilian Exclusive Economic Zone. Zerbini et al. (2004) conducted a pioneering study using mobile platforms to gather information regarding the presence of cetaceans along the south and the southeast coast. This quoted study shed some light on the anthropogenic impacts the recorded species might be exposed in the highly economically developed marine areas of Brazil. Yet, systematic studies regarding cetaceans in Brazil are still in their infancy.

The state of São Paulo (∼24°S) is the most developed throughout the Brazilian coast, hosting the largest port in Latin America (CODESP, 1992) and the largest terrestrial oil reservoir (Zanardi-Lamardo, Bícego & Weber, 2013), with the outcome of an intense traffic of large ships along its shore, where conflicts on the use of space with cetaceans have been turning into a cause of concern (Santos et al., 2010; Figueiredo et al., 2017). As of April 2020, 30 cetacean species were recorded along the shore of the quoted state (Santos et al., 2010; Santos & Figueiredo, 2016), considering year-round residents such as Guiana (*Sotalia guianensis*) and franciscana (*Pontoporia blainvillei*) dolphins, seasonal visitors such as the southern right (*Eubalaena australis*) and the humpback (*Megaptera novaeangliae*) whales, and strays from their original areas of distribution, such as several beaked whales and blackfish. Although several Marine Protected Areas (MPAs) were established throughout the coast of São Paulo state, almost none of the management plans considered cetacean richness, occurrence, distribution and species niche. Understanding niche segregation processes is critical in conservation ecology, particularly when investigating the ecology of species communities (Kiszka et al., 2011).

Peterson et al. (2011) defined niche as the set of ecological conditions needed for a species to maintain a population in a specific region, considering their impact on the available resources, the species they interact with, the environment and the habitat. Each species has a unique niche (Colwell & Rangel, 2009). The conservation of the niche is key to assess species potential distribution areas, historic distribution areas and alterations in their distributions due to climate change (Peterson, 2003; Soberón & Peterson, 2011; Warren, Glor & Turelli, 2008). The fundamental niche is the limit of the physiological tolerance to the environmental variables in which the species can maintain a positive population growth (Birch, 1953; Hutchinson, 1957). However, the fundamental niche can be larger than the limits allowed by the geographical space. The fundamental niche is then reduced to the potential niche according to the time and the available geographical space (Soberón & Peterson, 2011). The fundamental niche can also be reduced to the realized niche when biological interactions, such as competition, predation and resources consumption, are taken into consideration (Soberón & Peterson, 2011; Jackson & Overpeck, 2000).

The environmental features in which sympatric species occur are important to determine how they will coexist in both environmental and geographical space. In a study with different dolphin species of the genus *Stenella* in the western South Atlantic ocean, do Amaral et al. (2015) found that four species could exhibit niche partitioning and spatial segregation. This finding was similar to the differences in the distribution of three *Stenella* species along the Gulf of Mexico presented by Davis et al. (1998). The differences in the distribution patterns among *Stenella* species have been related not only to oceanographic conditions (do Amaral et al., 2015), but also to prey distribution and feeding preferences (Davis et al., 2005). However, in the Caribbean, dolphins of the genus *Stenella* did not show the same levels of niche partitioning, probably due to the low productivity levels in the Caribbean Sea (Barragán-Barrera et al., 2019).

The multi-species community of cetaceans recorded along the coast of São Paulo at southeastern Brazil offers an interesting opportunity to investigate the co-existence of several species in a limited geographical space shared with growing threats to their survival. The cetacean dataset gathered between 2012 and 2015 in the quoted area was analyzed with the following objectives: (1) to estimate the realized, fundamental and geographical niches and the niche overlap among the most common species found in the area; (2) to describe the habitat preference of the species inhabiting this area; (3) to provide insights on how this multi-species community may coexist. We hypothesized that those cetacean species may coexist due to niche partitioning, since the southeast coast of Brazil offers a wide range of different environmental features even in such limited extension of geographical space.

## Materials & Methods

### Study area

The study area includes approximately 600 km of shoreline from 23°20′S to 25°50′S. In this region the continental shelf can be more than 230 km wide (Castro & Miranda, 1998) and reach depths between 120 m and 180 m (Mahiques et al., 2010). The SBCS is influenced by three different water masses: Coastal Water (CW), Tropical Water (TW) and South Atlantic Central Water (SACW) (Campos, 1995; Castro & Miranda, 1998). With a NE-SW orientation, the coastline is very heterogeneous with wide and long beaches and the presence of estuaries south from 24°S, a transition area until 23°48′S and the presence of small bays, coves and islands in the northern section (Tessler et al., 2006).

Six MPAs with distinct regulations and management plans cover almost the whole extension of coastal waters up to 40 m deep: three state MPAs known as “Área de Proteção Ambiental Marinha Sul, Central, and Norte”; another two state units known as “Parque Estadual Marinho da Laje de Santos” and “Parque Estadual Marinho da Ilha Anchieta”, and two federal protected areas known as “Estação Ecológica Tupiniquins” and “Estação Ecológica Tupinambás”. In the latter, the Alcatrazes Archipelago has great relevance based on its historical relation to cetacean records.

### Oceanographic surveys

**Figure 1:**
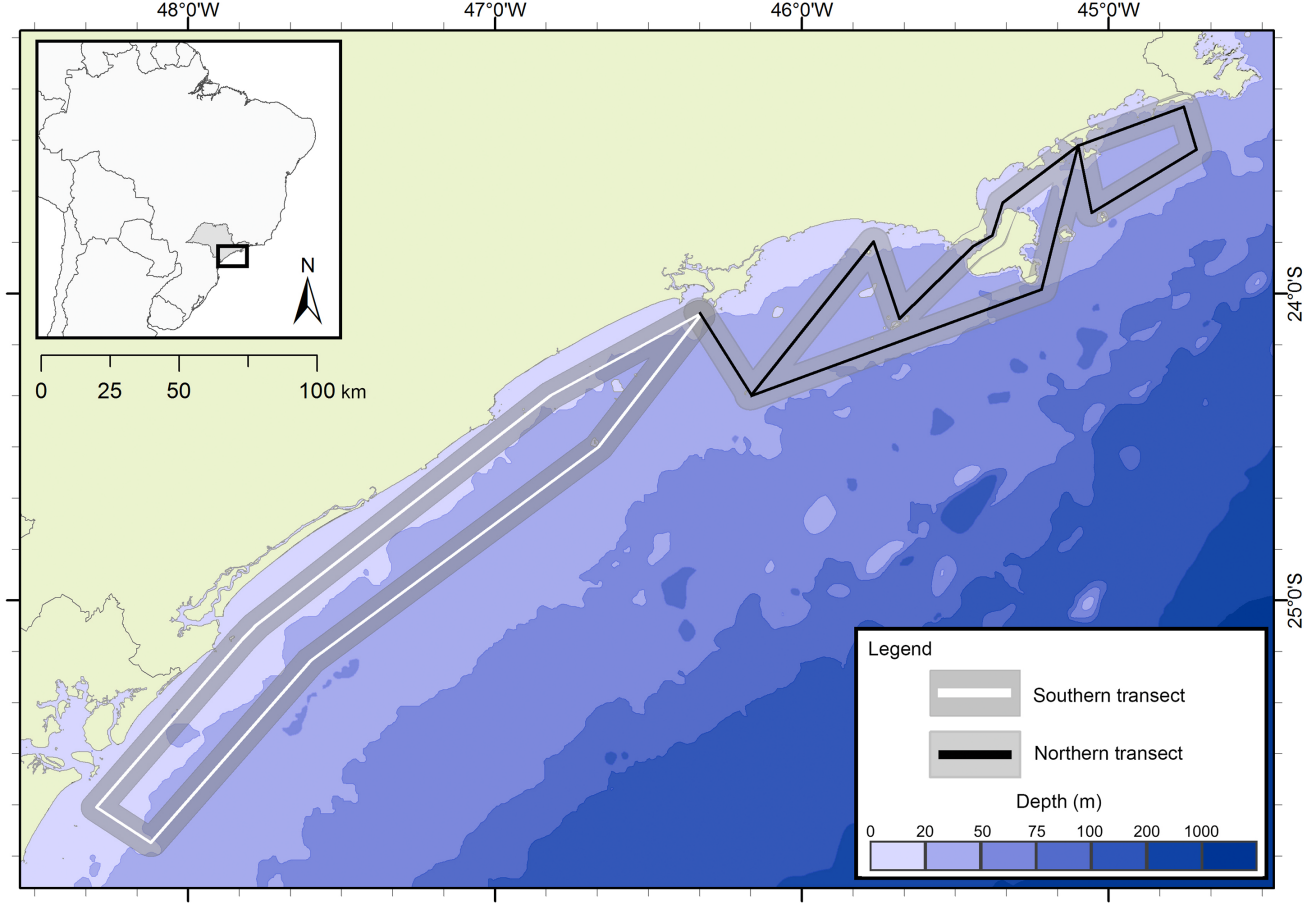
Study area and transects of the project “Occurrence, distribution and movement of cetaceans at São Paulo state coast”. The transects are divided in north (black) and south (white). The gray area represents the 5 km distance in which sightings could be made.

**Figure 2:**
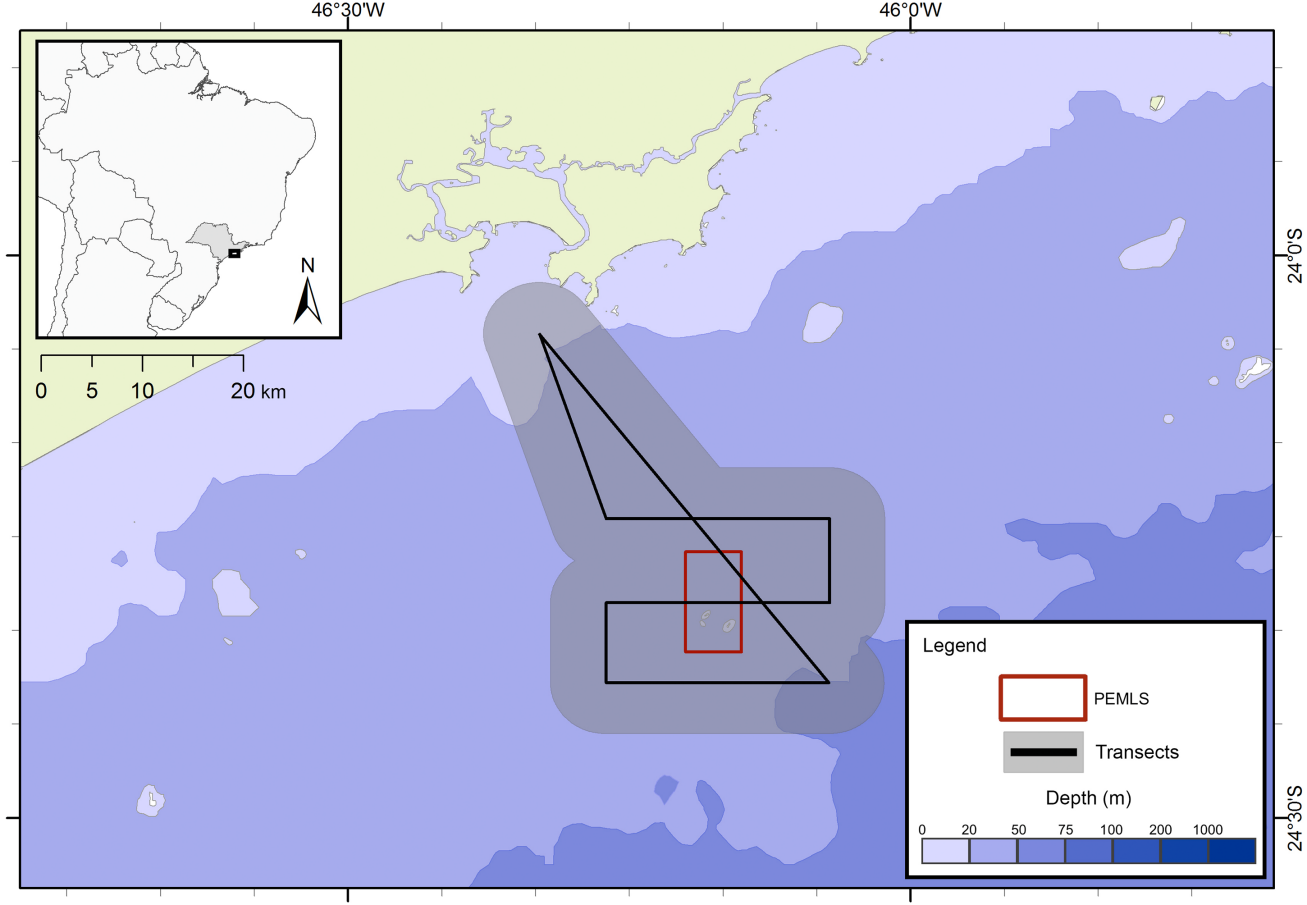
Transects for the PEMLS project. The gray area represents the 5 km distance in which sightings could be made and the red square indicates the limits of Parque Estadual Marinho da Laje de Santos (PEMLS).

The presence of cetaceans in the area was assessed during a series of oceanographic surveys from two different projects. The first project “Occurrence, distribution and movement of cetaceans at São Paulo state coast”, hereinafter referred to as FAP, covered the entire extension of the study area between 2012 and 2015, and was divided into north and south transects (Fig. 1) due to logistic reasons. For the second project, in this study referred as PEMLS (Fig. 2), the focus was to evaluate the presence of cetaceans in the area of the “Parque Estadual Marinho da Laje de Santos” between 2013 and 2015. Transects were based in Buckland et al. (2001) and Sutherland (2006) with a few modifications to cover the larger number of islands along the coast.

The same methodology was used in both projects. A 15 m motor powered boat traveling at 15 knots was used. Information regarding the GPS position of the boat, time, sea state (Beaufort scale), waves, visibility and glare were taken every 30 min, in the beginning and the end of transects, and when cetaceans were detected. In this later situation, the boat would approach in order to identify the species, estimate group size, and gather photographs of the dorsal fin of dolphins and balaenopterid whales, flukes of humpbacks and head callosities of right whales for individual identification. Digital reflex cameras with 400 mm lenses were used and photographs were then analyzed according to the criteria described by Santos & Rosso (2008) to compose a catalog for each reported species. This information was further used to assess individual movements in the study area (see Santos et al., 2019).

Instituto Chico Mendes de Conservação da Biodiversidade provided the permits to work with cetaceans in the research area, including fieldwork (permit number: 37206-1).

### Data analysis

The environmental data was obtained from global high-resolution rasters from MARSPEC (Sbrocco & Barber, 2013). The used variables were: depth of the seafloor (m), bathymetric slope (degrees), distance to shore (km), mean annual sea surface temperature (SST) (°C), annual variance in SST (°C), mean annual sea surface salinity (SSS) (psu) and annual variance in SSS (psu). These set of environmental variables are commonly used in other studies performed with cetaceans and are widely recognized to influence its distributions (see do Amaral et al., 2015, do Amaral et al., 2018; Barragán-Barrera et al., 2019) and references therein). ArcMapTM (Esri, http://www.esri.com) was used to extract the data for each sighting. Sightings without values regarding all environmental variables were excluded from the analysis.

For the species with 5+ sightings, univariate and multivariate analysis were performed to compare them in relation to the environmental variables measured at each sighting. Shapiro–Wilk test (shapiro.test, from stats package version 3.2.4) was used to assess if the data presented normal distribution. Kruskal-Wallis (kruskal.test from the same package) was used to test the null hypothesis that the distribution functions are the same among species (Zar, 1996). Dunn test (dunn.test from package dunn.test version 1.3.2) was used to determine which pair of species had a different distribution function (Dunn, 1964). The significance level *α* = 0.05 was used to all the previous analysis. Interspecies differences were further investigated through canonical discriminant analysis, which is a dimension-reduction technique related to principal component analysis (PCA) and canonical correlation. The methodology used in deriving the canonical coefficients parallels that of a one-way MANOVA. MANOVA tests for equality of the mean vector across class levels. Canonical discriminant analysis finds linear combinations of the quantitative variables that provide maximal separation among classes or groups. Given a classification variable and several quantitative variables, the CANDISC procedure derives canonical variables, linear combinations of the quantitative variables that summarize between-class variation in much the same way that principal components summarize total variation (Sas Institute Inc, 2017). The analyses were performed using standardized values of environmental variables through function scale (R Core Team, 2018) and canonical discriminant analysis was performed through candisc package (Friendly & Fox, 2020). Tests were performed in R v. 3.5.2 (R Core Team, 2018).

The species niche was estimated with the computational program NicheA, which uses scenopoetics variables to estimate Grinnellian niches (Qiao et al., 2016). Scenopoetic variables are non-interactive and, therefore, not affected by the species. NicheA uses the occurrence spots of the species and the three main components of the PCA of the environmental data to determine for each species the realized niche and fundamental niche as convex polyhedrons and ellipsoids in the environmental space, respectively. The fundamental niche was then transferred to the geographical space, indicating the closest areas to the centroid of the niche as the ones with adequate condition to the occurrence of the species. The volume and overlap of the fundamental and realized niche were calculated for every species pair, both in the environmental and geographic space.

## Results

**Figure 3:**
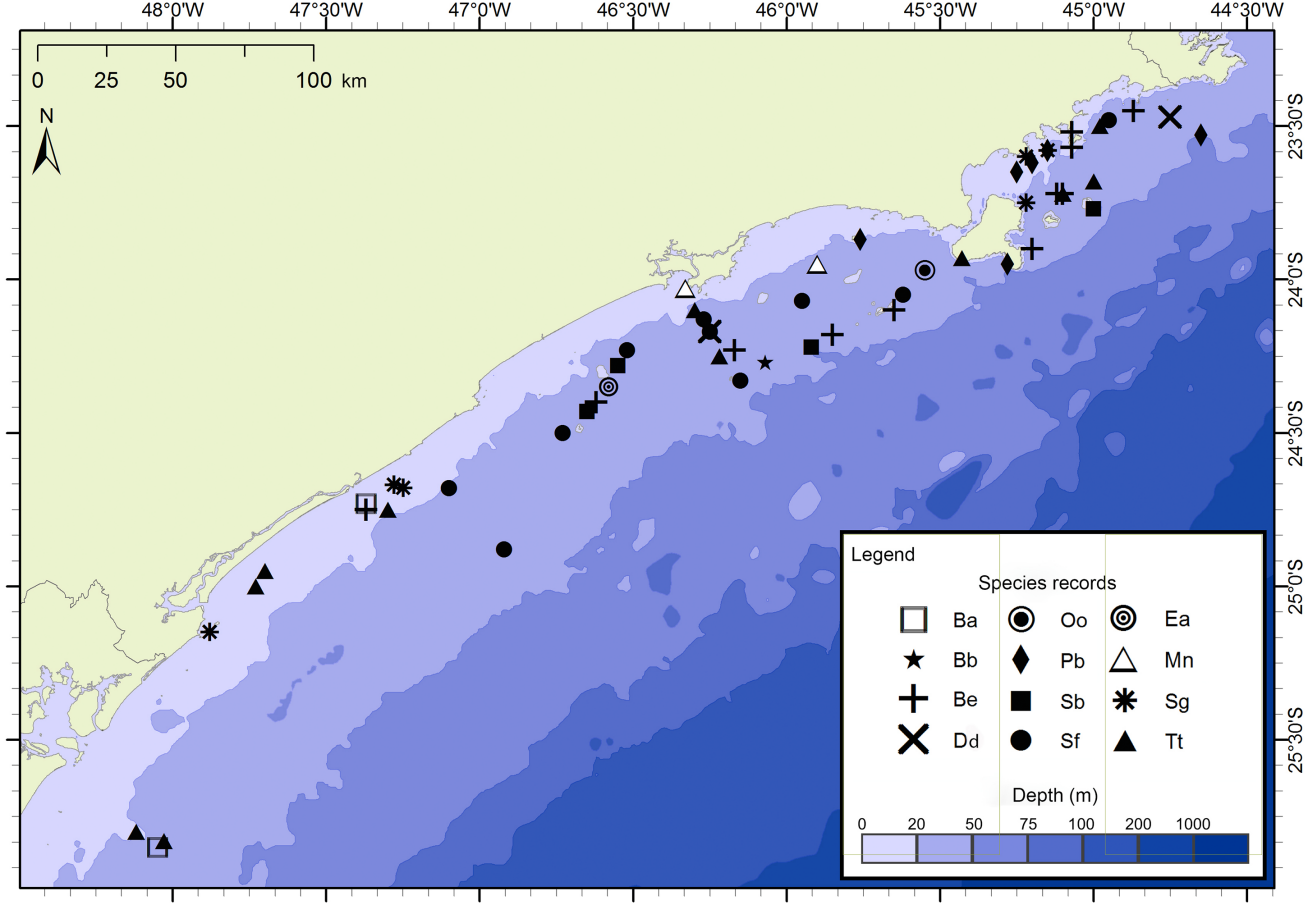
Sightings made in the 17 surveys of the FAP project between December 2012 and July 2015 along São Paulo State coast, Brazil. Species: Ba–*Balaenoptera acutorostrata*; Bb –*Balaenoptera bonaerensis*; Be –*Balaenoptera edeni*; Dd–*Delphinus delphis*; Ea–*Eubalaena australis*; Mn –*Megaptera novaeangliae*; Oo–*Orcinus orca*; Pb–*Pontoporia blainvillei*; Sb –*Steno bredanensis*; Sf–*Stenella frontalis*; Sg–*Sotalia guianensis*; Tt –*Tursiops truncatus*.

**Figure 4:**
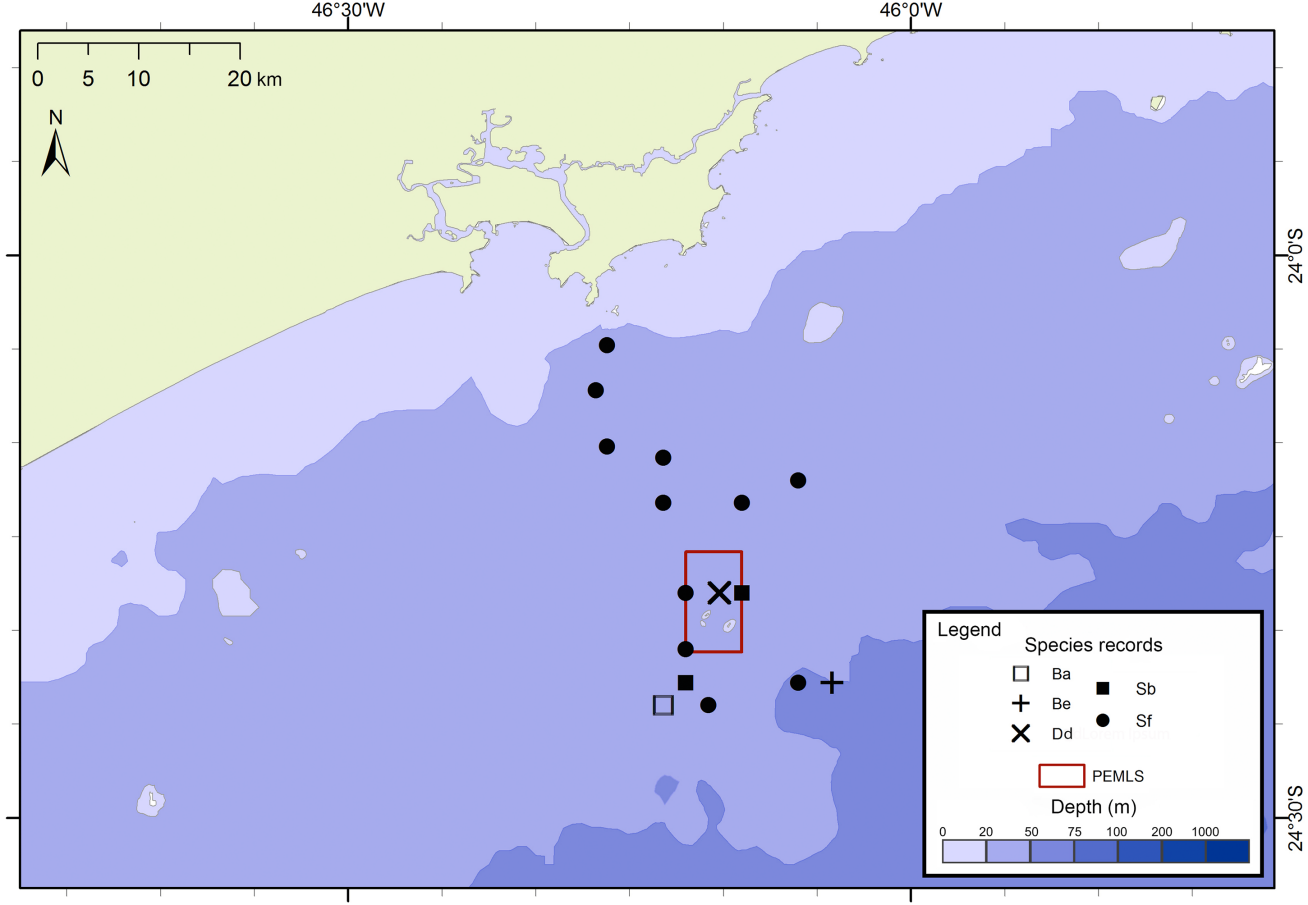
Sightings made in the 24 surveys of the PEMLS project between June 2013 and June 2015 along São Paulo State coast, Brazil. Species: Ba–*Balaenoptera acutorostrata*; Be –*Balaenoptera edeni*; Dd–*Delphinus delphis*; Sb–*Steno bredanensis*; Sf –*Stenella frontalis*.

The project FAP rendered 17 surveys in 50 days between December 2012 and July 2015, covering approximately 8,826 km of navigated transects. A total of 59 cetacean groups were reported, including five species of baleen whales and seven of toothed whales (Fig. 3). With an average of 1.3 sightings/day, the north sector had a higher sighting daily rate than the south sector with 1.0 sightings/day. The commonest species were Atlantic spotted dolphins, *Stenella frontalis*, the common bottlenose dolphin, *Tursiops truncatus* and the Bryde’s whale, *Balaenoptera edeni*, with 11 sightings each. The project PEMLS rendered 24 surveys between June 2013 and June 2015, covering approximately 3,346 km of transects (see supplementary materials). With an average of 0.8 sightings per survey, a total of 18 groups of cetaceans were sighted, with three baleen whale species and two toothed whales (Fig. 4). The Atlantic spotted dolphin was the species with the highest presence, representing 67% of all sightings.

Information regarding each sighting and the values of each environmental variable used in the analysis is presented as supplementary material. Sightings were made on depths ranging from 5 m to 76 m, between 1 km and 49 km from the shore and bathymetric slope between 0 and 7 degrees. Mean annual SST ranged between 22.9 °C and 24.0 °C (average 23.6 °C), and SSS varied between 34.3 and 35.8 (average 35.3), while annual variance ranged from 4.6 °C and 7.3 °C for SST, and from 0.6 to 1.2 for SSS.

Considering all surveys, the commonest species were *S. frontalis* (*n* = 23), *T. truncatus* (*n* = 11), *B. edeni* (*n* = 11), *P. blainvillei* (*n* = 7), *S. guianensis* (*n* = 6) and the rough-toothed dolphin, *Steno bredanensis* (*n* = 6). For the PEMLS project, the sighting rate of the Atlantic spotted dolphin was 0.0036 sightings/km. Considering the sightings in project FAP, the sighting rate of *S. frontalis* was 0.0037 sightings/km inside the area covered by the PEMLS surveys, and 0.001 sightings/km in the remaining area.

**Figure 5:**
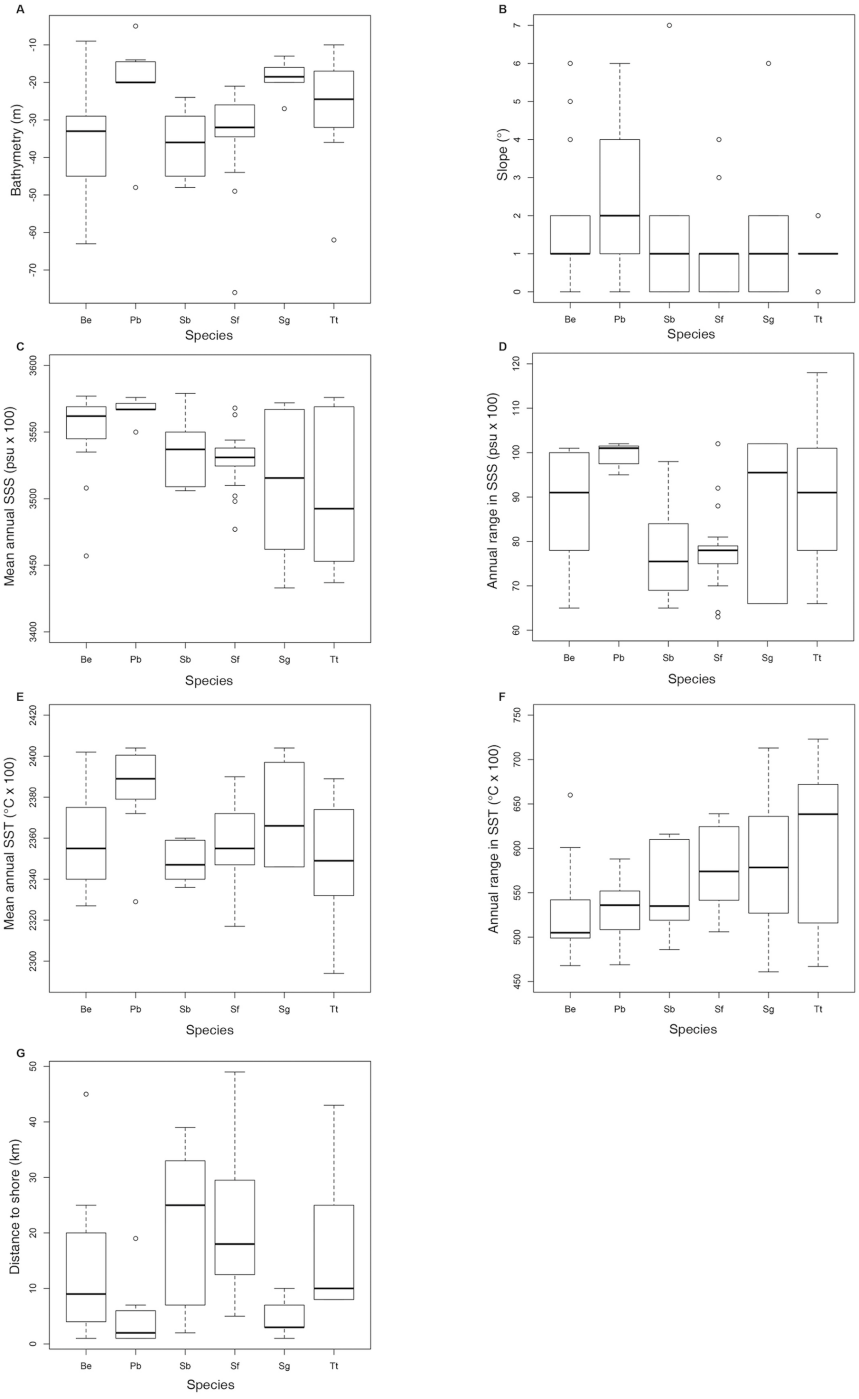
Boxplot with the data distribution of the six commonest species sighted along the southeastern Brazilian coast between 2012 and 2015. (A) Bathymetry (m); (B) Slope (^*o*^); (C) Mean annual SSS (psu ×100); (D) Annual range in SSS (psu ×100); (E) Mean annual SST (^*o*^*C* × 100); (F) Annual range in SST (^*o*^*C* × 100); (G) Distance to shore (km). Species: Be –*Balaenoptera edeni*; Pb –*Pontoporia blainvillei*; Sb–*Steno bredanensis*; Sf–*Stenella frontalis;* Sg –*Sotalia guianensis;* Tt–*Tursiops truncatus*.

Shapiro–Wilk test indicated that the majority of the variables did not show normal distribution. Therefore, non-parametric tests were used for further analysis. Kruskal-Wallis and Dunn test (Table 1) indicated that some pairs were significantly different regarding the analyzed environmental variables. In general, boxplot graphics (Fig. 5) showed that *S. bredanensis* and S. *frontalis* were recorded in deeper and farthest to shore waters, while *P. blainvillei* and *S. guianensis* were recorded in shallow and closest to shore waters. *S. guianensis* and *T. truncatus* were recorded in waters with low mean annual SSS, probably due to their presence closer to estuaries.

**Table 1:** Results of Kruskal–Wallis (KW) and Dunn tests. For the KW test, values in black indicate *p* < 0.05 and the rejection of the null hypothesis that all populations have the same distribution function. For the Dunn test, presented for each pair of species, values in bold indicate *p* < 0.05 and the pairs that differ regarding the variable tested.

Variable	Depth	Distance to shore	B. slope	Mean ann. SSS	Annual var. SSS	Mean ann. SST	Annual var. SST
KW test	**X**^**2**^**= 20.94**	**X**^**2**^**= 15.69**	X^2^ = 4.59	**X**^**2**^**= 15.69**	X^2^ = 1.04	X^2^ = 9.17	**X**^**2**^**= 13.16**
Be–Pb	*Z* = − 2.45	*Z* = 1.96	*Z* = − 0.73	*Z* = − 1.03	*Z* = 0.20	*Z* = − 1.94	*Z* = − 0.45
Be–Sb	*Z* = 0.59	*Z* = − 1.00	*Z* = 0.36	*Z* = 0.99	*Z* = − 0.49	*Z* = 0.93	*Z* = − 0.93
Pb–Sb	*Z* = 2.59	*Z* = − 2.54	*Z* = 0.93	*Z* = 1.75	*Z* = − 0.60	*Z* = 2.46	*Z* = − 0.44
Be–Sf	*Z* = − 0.09	*Z* = − 1.86	*Z* = 1.43	*Z* = 2.48	*Z* = − 0.43	*Z* = 0.13	** Z = -2.93**
Pb–Sf	*Z* = 2.59	**Z = -3.62**	*Z* = 1.93	** Z = 3.12**	*Z* = − 0.56	*Z* = 2.21	*Z* = − 1.87
Sb–Sf	*Z* = − 0.70	*Z* = − 0.33	*Z* = 0.69	*Z* = 0.82	*Z* = 0.20	*Z* = − 0.90	*Z* = − 1.23
Be–Sg	*Z* = − 2.64	*Z* = 1.75	*Z* = 0.41	*Z* = 1.56	*Z* = 0.10	*Z* = − 0.85	*Z* = − 1.75
Pb–Sg	*Z* = − 0.28	*Z* = − 0.10	*Z* = 0.97	*Z* = 2.25	*Z* = − 0.08	*Z* = 0.88	*Z* = − 1.17
Sb–Sg	**Z = -2.77**	*Z* = 2.35	*Z* = 0.04	*Z* = 0.49	*Z* = 0.51	*Z* = − 1.52	*Z* = − 0.70
Sf–Sg	**Z = -2.78**	** Z = 3.29**	*Z* = − 0.63	*Z* = − 0.20	*Z* = 0.43	*Z* = − 1.01	*Z* = 0.34
Be–Tt	*Z* = − 1.57	*Z* = − 0.65	*Z* = 0.63	*Z* = 2.32	*Z* = 0.34	*Z* = 0.78	** Z = -2.80**
Pb–Tt	*Z* = 0.99	*Z* = − 2.42	*Z* = 1.23	**Z = 2.96**	*Z* = 0.10	*Z* = 2.51	*Z* = − 1.96
Sb–Tt	*Z* = − 1.85	*Z* = 0.43	*Z* = 0.17	*Z* = 0.94	*Z* = 0.74	*Z* = − 0.25	*Z* = − 1.40
Sf–Tt	*Z* = − 1.66	*Z* = 0.98	*Z* = − 0.61	*Z* = 0.30	*Z* = 0.77	*Z* = 0.75	*Z* = − 0.42
Sg–Tt	*Z* = 1.25	*Z* = − 2.20	*Z* = 0.13	*Z* = 0.40	*Z* = 0.18	*Z* = 1.45	*Z* = − 0.61

**Notes.**

Species Be *Balaenoptera edeni* Pb *Pontoporia blainvillei* Sb *Steno bredanensis* Sf *Stenella frontalis* Sg *Sotalia guianensis* Tt *Tursiops truncatus*

**Figure 6:**
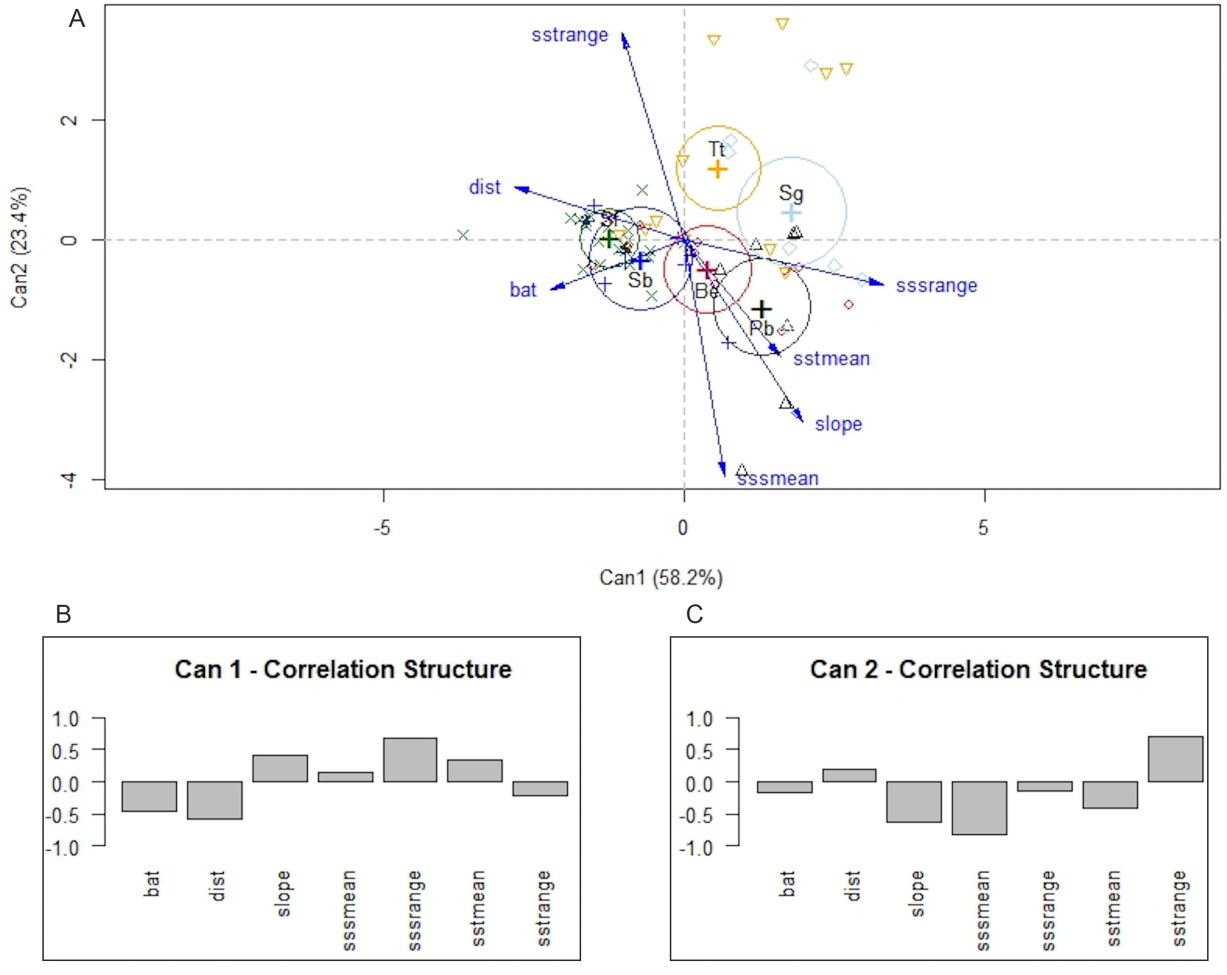
Canonical discriminant analysis separating species according to their environmental requirements in the study area. In (A) a plot of the first two canonical variables of canonical discriminant analysis, showing that canonical axis 1 discriminates species mainly in relation to coastal and offshore habitats. (B) and (C) structure correlations associated with canonical axis 1 and 2, respectively, representing the approximate correlations between the canonical variables and depth (bat), distance to shore (dist), slope, annual mean of SSS (sssmean), annual range in SSS (sssrange), annual mean of SST (sstmean), and annual range in SST (sstrange). Species: Be –*Balaenoptera edeni*; Pb–*Pontoporia blainvillei*; Sb–*Steno bredanensis*; Sf –*Stenella frontalis*; Sg–*Sotalia guianensis*; Tt–*Tursiops truncatus*.

Differences among species were also detected with MANOVA and canonical linear discriminant function analysis. The null hypothesis of equal mean vectors was rejected in the MANOVA (Wilks’ *λ* = 0.19895, *P* < 0.0001). The first two canonical variables accounted for 81.6% of the total variability. Likelihood ratio tests indicated that the first three canonical variables were significant (*P* < 0.0001, *P* < 0.001, and *P* < 0.5, respectively). The first canonical variable, *Can1*, showed that the linear combination of the centered variables separates the species most effectively, where *Can1 = −* 0.46 × depth −0.58 × distance to shore + 0.41 × bathymetric slope + 0.13 × mean annual SSS + 0.69 × annual variance in SSS + 0.33 × mean annual SST −0.23 × annual variance in SST. In summary, the structure correlations indicated that low values of annual range in SSS, distance to shore, and depth were associated with positive values of the first canonical variable, whereas low values of annual mean of SSS, and higher annual range in SST were associated with positive values of the second canonical variable (Fig. 6). Note that *P. blainvillei* and *S. guianensis* were clearly separated from *S. bredanensis* and *S. frontalis*, along canonical axis 1. Therefore, the separation among groups along canonical axis 1 supports the importance of distance to shore and depth in habitat partitioning in study area.

The volume for each ellipsoid (fundamental niche) and convex polyhedron (realized niche), and the overlap among species are presented in Table 2. The rough-toothed dolphin presented the smallest volume, while the Bryde’s whale showed the largest volume. There was overlap among all species except for *S. bredanensis* - *S. guianensis* for the environmental and realized niche, and *S. bredanensis* - *P. blainvillei* for the realized niche. Considering the volume, the highest overlaps regarding the fundamental niche were between *B. edeni* - *P. blainvillei* and *B. edeni* - *S. frontalis*. For the realized niche the largest overlaps were between *B. edeni* - *S. frontalis* and *B. edeni* - *T. truncatus*.

**Figure 7:**
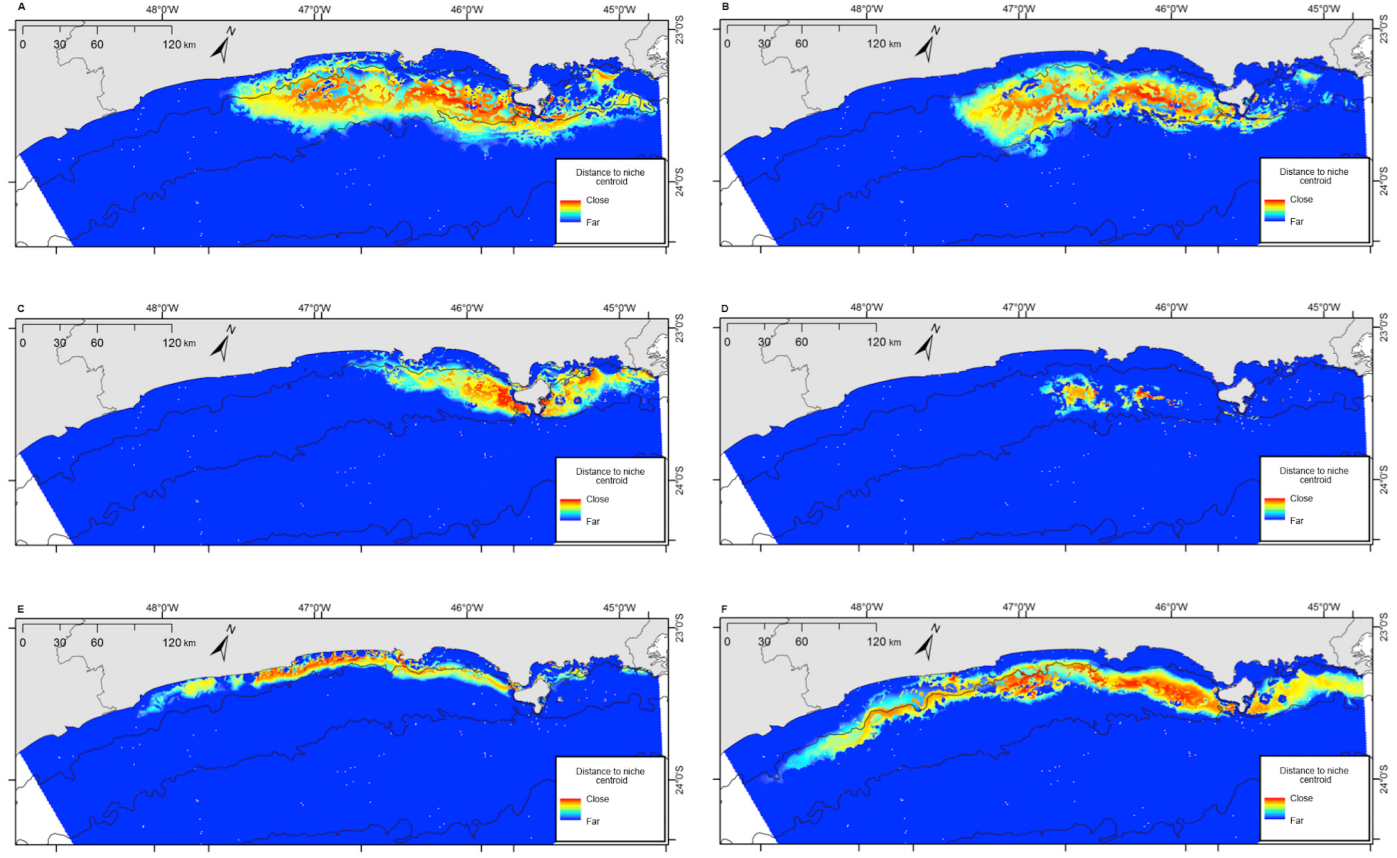
Projection of the fundamental niche in the geographic space for the six species most frequently sighted along the Brazilian southeastern coast between 2012 and 2015. Warmer colors indicate points closer to the centroid of the niche and therefore closer to the estimated ideal niche. (A) *Balaenoptera edeni*; (B) *Stenella frontalis*; (C) *Pontoporia blainvillei*; (D) *Steno bredanensis*; (E) *Sotalia guianensis*; (F) *Tursiops truncatus*.

**Figure 8:**
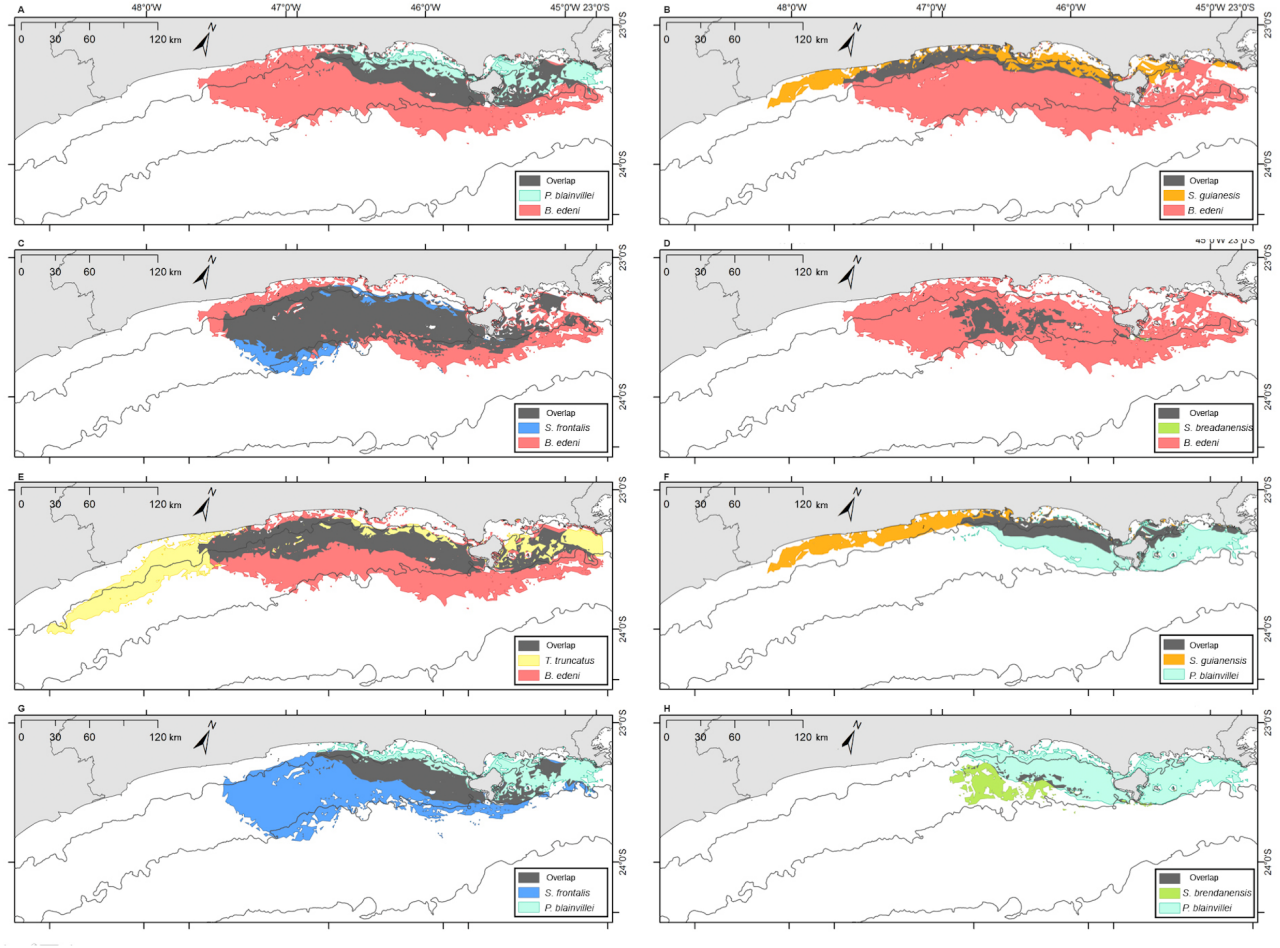
Overlap of the geographic niche for every pair of species of the six species most frequently sighted along the Brazilian southeastern coast between 2012 and 2015. Overlap area is shown in grey. (A) *P. blainvillei* and *B. edeni*; (B) *S. guianensis* and *B. edeni*; (C) *S. frontalis* and *B. edeni*; (D) *S. bredanensis* and *B. edeni*; (E) *T. truncatus* and *B. edeni*; (F) *S. guianensis* and *P. blainvillei*; (G) *S. frontalis* and *P. blainvillei*; (H) *S. bredanensis* and *P. blainvillei*.

**Figure 9:**
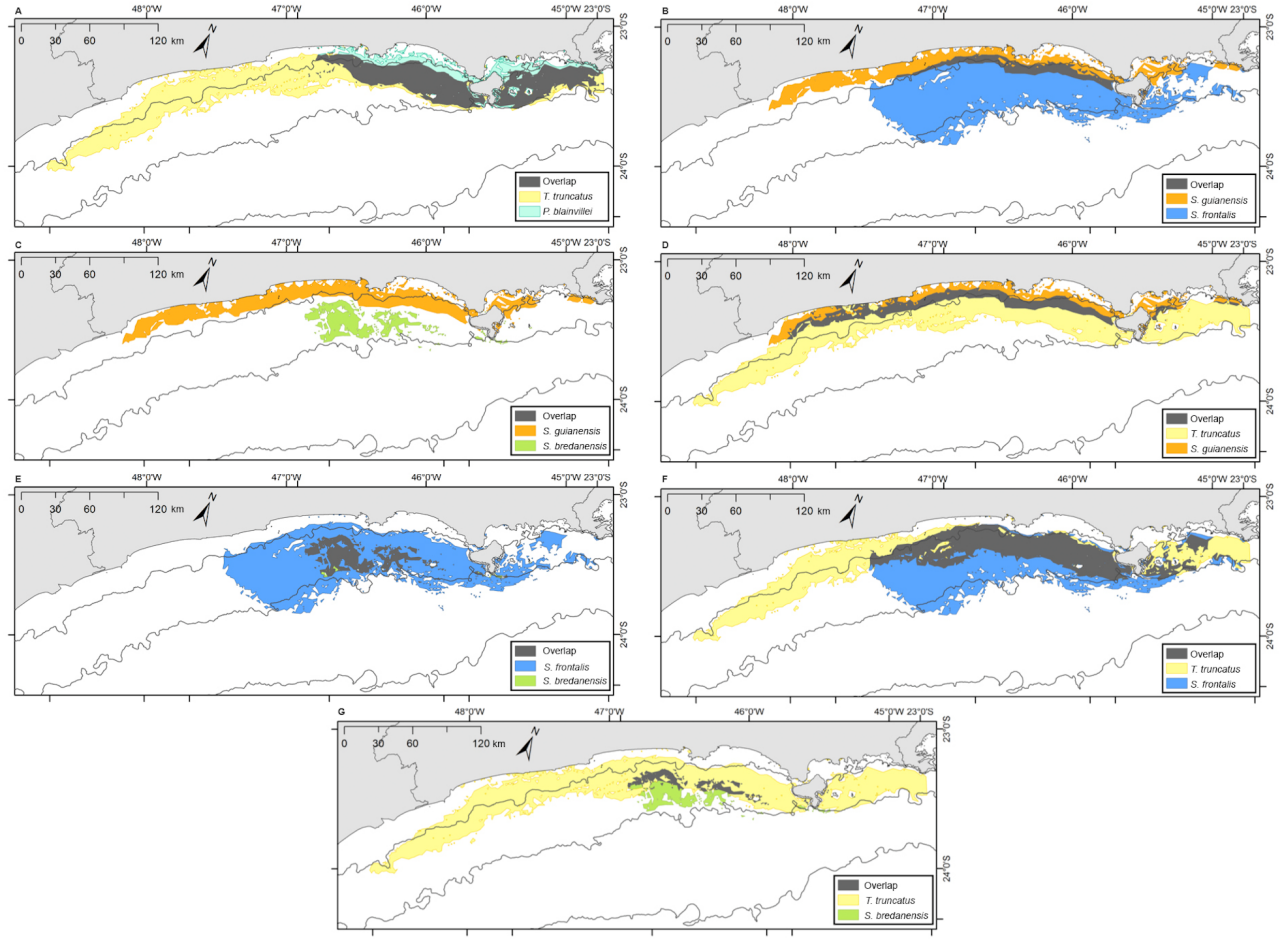
Overlap of the geographic niche for every pair of species of the six species most frequently sighted along the Brazilian southeastern coast between 2012 and 2015. Overlap area is shown in grey. (A) *T. truncatus* and *P. blainvillei*; (B) *S. guianensis* and *S*. *frontalis*; (C) *S. guianensis* and *S. bredanensis*; (D) *T. truncatus* and *S . guianensis*; (E) *S*. *frontalis* and *S. bredanensis*; (F) *T. truncatus* and *S. frontalis*; (G) *T. truncatus* and *S. bredanensis*.

Bryde’s whales’ potential distribution area covered the largest range. Bottlenose dolphins covered the longest extension along the coastline, while rough-toothed dolphins showed the most restricted potential distribution area among the six evaluated species (Fig. 7). Rough-toothed dolphins showed 99% of their potential distribution area overlapping the Bryde’s whale, 98% overlapping the Atlantic spotted dolphin and no overlap with the Guiana dolphin potential distribution area (Figs. 8 and 9 and Table 3).

## Discussion

The 12 cetacean species reported in this study were among the 30 species previously described to the surveyed area (Santos et al., 2010; Santos & Figueiredo, 2016), with six of these representing the coastal commonest species: one baleen whale and five toothed whales. Sightings and potential distribution areas described by the models for each surveyed species are in accordance with literature (Moreno et al., 2005; do Amaral et al., 2015; do Amaral et al., 2018). The northern sector of the study, between Ubatuba and Santos, presented the highest number of sightings. Compared to the southern sector, the northern shore shows the highest variability in environmental conditions and the higher abundance of islands, which may attract cetaceans searching for prey. Besides, the heterogeneity of the northern shore habitats may allow the co-occurrence of species with different environmental requirements.

**Table 2:** Values of fundamental (FN) and realized (RN) niche volumes and niche overlap between pairs of species calculated with NicheA considering the sightings reported along the Brazilian southeastern coast between 2012 and 2015.

Species	Be	Pb	Sb	Sf	Sg	Tt	FN	RN
Be	**–**	0.41	0.06	0.72	0.13	0.53	**7.11**	**1.96**
Pb	2.42	–	0	0.03	0.17	0.08	**3.55**	**0.58**
Sb	0.43	0.12	–	0.08	0	0.01	**0.61**	**0.09**
Sf	3.26	1.23	0.45	–	0.01	0.31	**4.33**	**0.10**
Sg	1.75	1.54	0	0.34	–	0.06	**4.78**	**0.95**
Tt	2.10	1.31	0.16	1.42	0.77	**–**	**6.12**	**1.57**

**Notes.**

Species Be *Balaenoptera edeni* Pb *Pontoporia blainvillei* Sb *Steno bredanensis* Sf *Stenella frontalis* Sg *Sotalia guianensis* Tt *Tursiops truncatus*

Considering the six commonest sighted species, it is important to highlight the new discoveries from the presented investigation.

### S. guianensis

*S. guianensis* has a continuous distribution along the coast from Honduras (14°N) to Florianópolis, southern Brazilian coast (27°S) (Crespo et al., 2010). They are usually reported in estuaries, bays and coastal sheltered areas, with most sightings reported up to 25 m deep, and apparently limited to 50 m of depth (da Silva et al., 2010). Along the coast of São Paulo State, knowledge of the species has been focused on long-term studies conducted with a resident population in a protected estuary in the southern sector, the Cananeia estuary (Santos et al., 2019). These were the first efforts directed towards gathering data on *S. guianensis* along local coastal waters. The potential distribution area described in this study shows a clear preference for shallow waters and the 20 m isobath as an average limit for the species distribution along the southeastern Brazilian coast.

### P. blainvillei

*P. blainvillei* also has a coastal distribution (do Amaral et al., 2018), with most of the sightings reported between 8 and 15 m deep, with limits at the 30 m isobath for sightings, and 35 m for incidental captures (Bordino et al., 2002). Most of the sightings reported here were made inside those limits, except for two groups sighted at 35 m and 44 m deep. The mean depth of the presented sightings was also higher than the described limits for the species. The absence of sightings along the central and the southern coasts of the surveyed coast could be explained by the water transparency –darker in the quoted subareas -, the cryptic behavior of the species, and its preference to use shallower waters close to the shore which were not completely surveyed due safety reasons.

### S. frontalis

Throughout its range, *S. frontalis* is mainly reported in the continental shelf up to 1,000 m of depth across tropical and subtropical waters in the Atlantic Ocean (Herzing & Perrin, 2017). Along the Brazilian coast, the species is recorded mainly from 18 to 34°S and northern of 6°S (Moreno et al., 2005; do Amaral et al., 2015). The central region of São Paulo State coast showed the highest number of sightings of *S. frontalis*, especially in the area surrounding the PEMLS. This MPA is located in the central part of the potential distribution area estimated by the model for this species. The present and a previous study (Santos, Figueiredo & Van Bressen, 2017) show a year-round presence of Atlantic spotted dolphins along the coast of São Paulo, rendering an excellent opportunity for longitudinal investigations.

### T. truncatus

The bottlenose dolphin was the third most common species in this study and had the second largest potential distribution area, with sightings occurring throughout the entire surveyed area. The species is known to have a continuous distribution along the Brazilian coastal waters and estuaries (Lodi et al., 2016) in depths ranging from 1.6 to 50 m (Laporta et al., 2016). Particularly since 1990, longitudinal studies have been conducted with common bottlenoses in estuaries and coastal waters of southern Brazil (Simões-Lopes & Fábian, 1999; Fruet et al., 2015). From Santa Catarina State (∼27°S) to lower latitudes, *S. guianensis* occupies shallower waters (da Silva et al., 2010), meanwhile *T. truncatus* are found in slightly deeper waters (up to 50 m). The distribution patterns described by the models in the present study clearly show the Guiana dolphin potential distribution area closer to the shore, the bottlenose dolphin found in deeper waters, and the overlap between them found close to the 20 m isobath.

### B. edeni

Sightings of *B. edeni* in the study area are in agreement with literature, as they are reported year round along the southeastern Brazilian coast, with a higher number of sightings among spring and summer months when they are in search of preys in shallower waters (Zerbini et al., 1997; Siciliano et al., 2004; Santos et al., 2010; Gonçalves, Augustowski & Andriolo, 2016; Santos et al., 2019). The geographical areas indicated as closer to the centroid of the species niche, like São Sebastião Island and the Alcatrazes archipelago, could be related to high biological productivity. Jefferson, Webber & Pitman (2008) and Kato & Perrin (2009) suggested that Bryde’s whales have a preference for areas with higher productivity in tropical and subtropical basins. Tardin et al. (2019) showed a positive correlation between *B. edeni* and high concentrations of chl-a in depths between 30 and 60m in southeast Brazil.

### S. bredanensis

The rough-toothed dolphin is known for its presence in deep and oceanic tropical and subtropical waters, with common records in shallow waters of the southeast coast of Brazil (Lodi, 1992; Jefferson, 2017; Santos et al., 2019). Evidences of site and group fidelity were previously observed in the surveyed area (Santos et al., 2019). The small geographical area for the potential niche indicates a possible restriction in the use of area and also for the environmental requirements for the investigated stock. As species with restricted distribution are more vulnerable to human impact (Gaston & Fuller, 2009), it is important to further understand their life history and environmental requirements in the coast of São Paulo state.

### Niche overlap

The ecological niche models (ENM) presented in this study indicated a clear overlap between species niche and their potential distribution area. With largest niche volume and potential distribution area, *B. edeni* and *S. frontalis* are known to occupy areas that expand further than the SBCS. They also presented the largest niche overlap, not only between them, but also with other species (Figs. 7–9). The exception is the overlap with *S. guianensis*. The Guiana dolphin occupies areas closer to the shore and showed overlaps with *P. blainvillei*, and *T. truncatus*, and no overlap with *S. bredanensis*.

The use of ENMs is relevant when considering species with wide distribution like *S. frontalis* and *T. truncatus*, as it allows the evaluation of precise areas of occurrence. This information can be used to further understand the biogeography of those species, as well as to investigate their trophic ecology, like prey distribution according to predators.

**Table 3:** Values of the geographic niche area (km^2^) and niche overlap between pairs of species for the sightings made along the Brazilian southeastern coast between 2012 and 2015. Values in bold indicate the total area for each species.

Species	Be	Pb	Sb	Sf	Sg	Tt
Be	**12,969**	–	–	**-**	**-**	**-**
Pb	3,230	**5,318**	**-**	**-**	**-**	**-**
Sb	1,316	204	**1,333**	**-**	**-**	**-**
Sf	8,740	3,082	1,288	**10,188**	**-**	**-**
Sg	1,694	2,001	0	1,099	**4,090**	**-**
Tt	5,689	3,961	518	4,854	2,080	**10,072**

**Notes.**

Species Be *Balaenoptera edeni* Pb *Pontoporia blainvillei* Sb *Steno bredanensis* Sf *Stenella frontalis* Sg *Sotalia guianensis* Tt *Tursiops truncatus*

It was expected that the franciscana dolphin would have a similar geographical area to the Guiana dolphin, as they are both reported to use coastal and turbid waters (Santos et al., 2002). However, the species was only sighted in surveys in the northern sector and in higher depths than they are usually reported. The clearer waters of this region could have raised the probabilities of sightings. Therefore, the niche estimated and higher values of mean annual SST and SSS obtained here may be biased for the entire study area, since individuals from the Southern sector were not portrayed in the analysis due to lesser probabilities of sightings. Nonetheless, it is important to better evaluate the way distinct populations use the area for conservation purposes, as a genetic distinction in stocks was pointed out by Cunha et al. (2014). Franciscana dolphins are vulnerable to extinction with a high mortality provoked by incidental captures throughout its whole restricted distribution (Zerbini et al., 2018). Thus, any information on the biogeography of distinct stocks is relevant to establish management plans.

Besides predation of prey, the only species interaction observed was a mixed group of 12 bottlenose dolphins and one Bryde’s whale in December 2012. do Amaral et al. (2015) and Di Tullio et al. (2016) reported the presence of mixed groups of cetaceans along the Brazilian coast. The bottlenose dolphin was present in more than half of the groups reported by Di Tullio et al. (2016) in deeper waters off Southern Brazil. Aggressive behavior was reported between males of Atlantic spotted and bottlenose dolphins in the Bahamas (Herzing, Moewe & Brunnick, 2003). Bottlenose and Guiana dolphins had previously been observed in aggressive interactions ignited by the larger bottlenoses (Acevedo-Gutierez et al., 2005; Wedekin, Daura-Jorge & Simões-Lopes, 2004) in both limits of the distribution of *S. guianensis*. In a general sense, toothed whales tend to avoid direct competition by adopting different behaviors, diets and physiologic habits (Bearzi, 2005). These characteristics might explain the observed species co-occurrences in the surveyed area.

Niche overlap is also related to geographical overlap (Warren, Glor & Turelli, 2008). Partitioning resources can reduce competition among species that occupy the same area (Roughgarden, 1976). This could explain the considerable niche and geographical overlap estimated for *S. frontalis*, *B. edeni* and *T. truncatus* (see Figs. 6–8; Tables 2 and 3). Another alternative is that the environment used by these species has abundant resources, therefore allowing them to coexist (Bearzi, 2005).

The presence of cetaceans close to several islands in the studied area might be related to the “island effect”, which means that oceanic islands present higher productivity than surrounding areas (Doty & Oguri, 1956). The areas including the main islands (e.g., Ilha Anchieta, Ilhabela, Alcatrazes, Laje de Santos) in this survey are close to the center of the niche centroid for *B. edeni*, *S. frontalis*, *P. blainvillei* and *S. bredanensis*. This result highlights the importance of these areas, which, in most occasions, are all included inside the limits of established MPAs. However, none of the published management plans considered the patterns of occurrence and distribution of cetaceans. Thus, we recommend that the results presented here should be considered in the next revision of these tools for nature conservation.

## Conclusions

The results suggest that *B. edeni, S. frontalis*, *T. truncatus*, *S. bredanensis*, *S. guianensis* and *P. blainvillei* sighted in the area of investigation exhibited some level of overlap both for the niche and the geographical area. They also presented some level of niche partitioning, suggesting they might occupy different positions in the environmental space, mainly due its topographic preferences and distance to shore. Therefore, this community of cetaceans can co-occur due to niche partitioning and the wide range of environmental features available in the area. These findings should be considered in the management plans of the established MPAs of southeast Brazil.

##  Supplemental Information

10.7717/peerj.10000/supp-1 Supplemental Information 1 Cetacean sightings along the coast of São Paulo state, southeastern Brazil, from 2012 to 2015, considering two distinct projects (FAP and PEMLS) Includes: date, species (SPP), sector (N–north; S –south, P–PEMLS project), latitude, longitude, group size, depth of the seafloor (m), distance to shore (km), bathymetric slope (degrees), mean annual SST (°C), annual variance in SST (°C), mean annual SSS (psu) and annual variance in SSS (psu). Species: Ba –*Balaenoptera ocutorostrata*; Bb –*Balaenoptera bonaerensis*; Be –*Balaenoptera edeni*; Dd–*Delphinus delphis*; Ea–*Eubalaena australis*; Mn –*Megaptera novaeangliae*; Oo–*Orcinus orca*; Pb–*Pontoporia blainvillei*; Sb –*Steno bredanensis*; Sf–*Stenella frontalis*; Sg–*Sotalia guianensis*; Tt –*Tursiops truncatus*

## Additional Information and Declarations

### Data Availability

The following information was supplied regarding data availability: The environmental data used in the analysis are available at www.marspec.org. The sighting data are available in Supplemental Material, including the environmental data extracted from MARSPEC.
