# Supplementary material for: Cetaceans along the southeastern Brazilian coast: occurrence, distribution and niche inference at local scale
Source: PeerJ. 2020 Oct 5;8:e10000. doi: 10.7717/peerj.10000 (PMC7543724; doi:10.7717/peerj.10000)
Supplement: Supplemental Information 1 — Includes: date, species (SPP), sector (N–north; S –south, P–PEMLS project), latitude, longitude, group size, depth of the seafloor (m), distance to shore (km), bathymetric slope (degrees), mean annual SST (°C), annual variance in SST (°C), mean annual SSS (psu) and annual variance in SSS (psu). Species: Ba –Balaenoptera ocutorostrata; Bb –Balaenoptera bonaerensis; Be –Balaenoptera edeni; Dd–Delphinus delphis; Ea–Eubalaena australis; Mn –Megaptera novaeangliae; Oo–Orcinus orca; Pb–Pontoporia blainvillei; Sb –Steno bredanensis; Sf–Stenella frontalis; Sg–Sotalia guianensis; Tt –Tursiops truncatus [file peerj-08-10000-s001.docx]

Cetacean sightings along the coast of São Paulo state, southeastern Brazil, from 2012 to 2015, considering two distinct projects (FAP and PEMLS), including: date, species (SPP), sector (N – north; S – south, P – PEMLS project), latitude, longitude, group size, depth of the seafloor (m), distance to shore (km), bathymetric slope (degrees), mean annual SST (°C), annual variance in SST (°C), mean annual SSS (psu) and annual variance in SSS (psu).

| Date | SPP | Sector | Lat | Long | Group size | Depth | Dist. shore | Slope | Mean SST | Var. SST | Mean SSS | Var. SSS |
| --- | --- | --- | --- | --- | --- | --- | --- | --- | --- | --- | --- | --- |
| 06/dec/12 | Be | N | -23.72 | -45.10 | 1 | -30 | 9 | 2 | 23.7 | 4.7 | 35.7 | 1.0 |
| 06/dec/12 | Tt | N | -23.72 | -45.10 | 12 | -30 | 9 | 2 | 23.7 | 4.7 | 35.7 | 1.0 |
| 06/dec/12 | Be | N | -23.72 | -45.12 | 1 | -30 | 9 | 2 | 23.8 | 4.7 | 35.7 | 1.0 |
| 07/dec/12 | Oo | N | -23.97 | -45.55 | 18 | -37 | 13 | 1 | 23.8 | 5.7 | 35.6 | 1.0 |
| 07/dec/12 | Be | N | -24.18 | -45.85 | 1 | -45 | 20 | 0 | 23.4 | 5.2 | 35.5 | 0.9 |
| 07/dec/12 | Sb | N | -24.22 | -45.92 | 20 | -45 | 28 | 1 | 23.4 | 5.2 | 35.5 | 0.8 |
| 12/jun/13 | Sf | N | -23.48 | -44.95 | 120 | -76 | 9 | 4 | 23.9 | 5.1 | 35.7 | 1.0 |
| 12/jun/13 | Be | N | -23.45 | -44.87 | 1 | -63 | 8 | 5 | 23.9 | 4.9 | 35.7 | 1.0 |
| 12/jun/13 | Sg | N | -23.75 | -45.22 | 180 | -27 | 3 | 2 | 23.9 | 4.6 | 35.7 | 1.0 |
| 13/jun/13 | Be | N | -23.90 | -45.20 | 2 | -40 | 3 | 1 | 23.7 | 5.0 | 35.8 | 1.0 |
| 31/jul/13 | Sf | P | -24.17 | -46.27 | 60 | -26 | 15 | 1 | 27.7 | 6.1 | 35.3 | 0.8 |
| 02/sep/13 | Ea | S | -24.35 | -46.58 | 2 | -26 | 15 | 1 | 23.7 | 6.1 | 35.3 | 0.8 |
| 02/sep/13 | Be | S | -24.40 | -46.62 | 1 | -27 | 19 | 0 | 23.6 | 6.1 | 35.1 | 0.7 |
| 02/sep/13 | Sb | S | -24.43 | -46.65 | 15 | -29 | 11 | 0 | 23.5 | 6.0 | 35.1 | 0.7 |
| 10/sep/13 | Tt | S | -24.95 | -47.70 | 30 | -29 | 7 | 0 | 23.5 | 6.1 | 35.1 | 0.7 |
| 10/sep/13 | Be | S | -24.75 | -47.37 | 1 | -10 | 9 | 1 | 23.3 | 6.7 | 34.4 | 0.8 |
| 10/sep/13 | Ba | S | -24.73 | -47.37 | 2 | -15 | 10 | 0 | 23.4 | 6.6 | 34.6 | 0.7 |
| 27/nov/13 | Sf | P | -24.30 | -46.20 | 40 | -32 | 31 | 0 | 23.5 | 5.4 | 35.3 | 0.8 |
| 27/nov/13 | Be | P | -24.38 | 46.07 | 2 | -50 | 45 | 1 | 23.3 | 5.1 | 35.5 | 0.8 |
| 28/nov/13 | Be | P | -24.38 | 46.07 | 2 | -50 | 45 | 1 | 23.3 | 5.1 | 35.5 | 0.8 |
| 28/jan/14 | Sf | N | -24.07 | -45.95 | 60 | -12 | 8 | 0 | 23.3 | 6.7 | 34.6 | 0.7 |
| 29/jan/14 | Be | N | -23.40 | -45.07 | 2 | -32 | 31 | 0 | 23.5 | 5.4 | 35.3 | 0.8 |
| 30/jan/14 | Be | N | -24.23 | -46.17 | 2 | -50 | 45 | 1 | 23.3 | 5.1 | 35.5 | 0.8 |
| 05/feb/14 | Sf | P | -24.35 | -46.20 | 30 | -32 | 36 | 1 | 23.4 | 5.4 | 35.4 | 0.8 |
| 10/feb/14 | Sf | S | -24.23 | -46.52 | 25 | -50 | 45 | 1 | 23.3 | 5.1 | 35.5 | 0.8 |
| 10/feb/14 | Tt | S | -24.75 | -47.30 | 15 | -30 | 28 | 1 | 23.7 | 5.7 | 35.4 | 0.9 |
| 25/feb/14 | Sf | S | -24.88 | -46.92 | 2 | -9 | 2 | 4 | 24.0 | 5.0 | 35.7 | 1.0 |
| 26/feb/14 | Tt | S | -25.80 | -48.12 | 35 | -35 | 25 | 1 | 23.6 | 5.5 | 35.4 | 0.8 |
| 26/feb/14 | Sg | S | -25.15 | -47.88 | 2 | -32 | 36 | 1 | 23.4 | 5.3 | 35.4 | 0.8 |
| 27/feb/14 | Sg | S | -24.68 | -47.25 | 40 | -24 | 18 | 0 | 23.7 | 6.3 | 35.1 | 0.7 |
| 01/apr/14 | Sf | P | -24.12 | -46.28 | 30 | -26 | 9 | 0 | 23.8 | 6.3 | 35.3 | 0.8 |
| 01/apr/14 | Sf | P | -24.38 | -46.10 | 250 | -49 | 43 | 1 | 23.3 | 5.1 | 35.4 | 0.8 |
| 01/apr/14 | Sf | P | -24.20 | -46.10 | 250 | -35 | 26 | 1 | 23.5 | 5.6 | 35.4 | 0.8 |
| 01/apr/14 | Sf | P | -24.22 | -46.22 | 8 | -33 | 21 | 0 | 23.6 | 5.7 | 35.3 | 0.8 |
| 07/may/14 | Pb | N | -23.62 | -45.20 | 2 | -17 | 14 | 1 | 23.4 | 6.6 | 34.6 | 0.7 |
| 07/may/14 | Pb | N | -23.58 | -45.15 | 4 | -44 | 49 | 1 | 23.2 | 6.3 | 35.0 | 0.6 |
| 07/may/14 | Sg | N | -23.58 | -45.15 | 14 | -17 | 34 | 1 | 23.0 | 7.2 | 34.5 | 1.2 |
| 07/may/14 | Pb | N | -23.53 | -44.65 | 4 | -18 | 3 | 0 | 23.5 | 7.1 | 34.3 | 0.9 |
| 08/may/14 | Tt | N | -23.68 | -45.00 | 80 | -19 | 10 | 0 | 23.5 | 6.2 | 34.7 | 0.7 |
| 08/may/14 | Bb | N | -24.27 | -46.07 | 1 | -26 | 9 | 0 | 23.8 | 6.3 | 35.3 | 0.8 |
| 08/may/14 | Sf | N | -24.33 | -46.15 | 120 | -49 | 43 | 1 | 23.3 | 5.1 | 35.4 | 0.8 |
| 24/jun/14 | Sf | P | -24.40 | -46.18 | 70 | -42 | 42 | 1 | 23.3 | 5.3 | 35.4 | 0.8 |
| 25/jun/14 | Ba | P | -24.40 | -46.22 | 2 | -40 | 41 | 1 | 23.3 | 5.4 | 35.4 | 0.8 |
| 31/jul/14 | Pb | N | -23.58 | -45.15 | 2 | -35 | 26 | 1 | 23.5 | 5.6 | 35.4 | 0.8 |
| 31/jul/14 | Be | N | -23.52 | -45.07 | 1 | -33 | 21 | 0 | 23.6 | 5.7 | 35.3 | 0.8 |
| 31/jul/14 | Sb | N | -23.77 | -45.00 | 25 | -15 | 5 | 0 | 23.9 | 5.1 | 35.7 | 1.0 |
| 19/aug/14 | Mn | S | -24.03 | -46.33 | 2 | -20 | 1 | 6 | 24.0 | 5.4 | 35.7 | 1.0 |
| 19/aug/14 | Sf | S | -24.68 | -47.10 | 40 | -20 | 1 | 6 | 24.0 | 5.4 | 35.7 | 1.0 |
| 20/aug/14 | Ba | S | -25.85 | -48.05 | 2 | -48 | 19 | 2 | 23.7 | 4.7 | 35.8 | 1.0 |
| 20/aug/14 | Tt | S | -25.83 | -48.03 | 17 | -36 | 8 | 1 | 23.7 | 4.7 | 35.8 | 1.0 |
| 21/aug/14 | Tt | S | -25.00 | -47.73 | 55 | -42 | 34 | 0 | 23.4 | 5.2 | 35.4 | 0.8 |
| 21/aug/14 | Sg | S | -24.67 | -47.28 | 30 | -33 | 35 | 3 | 23.4 | 5.3 | 35.4 | 0.8 |
| 11/nov/14 | Tt | N | -24.25 | -46.22 | 26 | -42 | 42 | 1 | 23.3 | 5.3 | 35.4 | 0.7 |
| 17/dec/14 | Tt | N | -24.10 | -46.30 | 65 | -40 | 41 | 1 | 23.3 | 5.4 | 35.4 | 0.7 |
| 18/dec/14 | Pb | N | -23.65 | -45.25 | 5 | -20 | 1 | 6 | 24.0 | 5.4 | 35.7 | 1.0 |
| 19/dec/14 | Pb | N | -23.95 | -45.28 | 2 | -16 | 1 | 6 | 24.0 | 5.4 | 35.7 | 1.0 |
| 19/dec/14 | Tt | N | -23.95 | -45.43 | 35 | -48 | 2 | 7 | 23.6 | 4.9 | 35.8 | 1.0 |
| 19/dec/14 | Be | N | -24.10 | -45.65 | 4 | -14 | 2 | 2 | 23.8 | 6.2 | 35.2 | 0.8 |
| 19/dec/14 | Sf | N | -24.13 | -46.27 | 200 | -21 | 18 | 1 | 23.5 | 6.4 | 34.8 | 0.6 |
| 15/jan/15 | Sf | P | -24.08 | -46.27 | 12 | -23 | 5 | 1 | 23.9 | 6.2 | 35.3 | 0.8 |
| 20/jan/15 | Sf | S | -24.68 | -47.10 | 250 | -26 | 43 | 1 | 22.9 | 7.3 | 34.6 | 1.2 |
| 03/feb/15 | Sb | S | -24.28 | -46.55 | 15 | -26 | 43 | 1 | 22.9 | 7.2 | 34.6 | 1.2 |
| 03/feb/15 | Sf | S | -24.50 | -46.73 | 200 | -11 | 11 | 1 | 23.3 | 6.7 | 34.4 | 0.8 |
| 10/feb/15 | Dd | N | -24.17 | -46.25 | 12 | -16 | 7 | 1 | 23.5 | 6.4 | 34.6 | 0.7 |
| 26/feb/15 | Sb | P | -24.30 | -46.15 | 15 | -34 | 33 | 1 | 23.4 | 5.4 | 35.4 | 0.8 |
| 19/may/15 | Sb | P | -24.38 | -46.20 | 50 | -38 | 39 | 2 | 23.4 | 5.3 | 35.4 | 0.8 |
| 20/may/15 | Dd | P | -24.30 | -46.17 | 7 | -31 | 32 | 1 | 23.5 | 5.4 | 35.4 | 0.8 |
| 26/may/15 | Sf | P | -24.18 | -46.22 | 50 | -30 | 17 | 0 | 23.7 | 6.0 | 35.3 | 0.8 |
| 08/jun/15 | Sf | P | -24.22 | -46.15 | 20 | -36 | 25 | 1 | 23.6 | 5.5 | 35.4 | 0.8 |
| 08/jun/15 | Sf | P | -24.22 | -46.22 | 120 | -33 | 21 | 0 | 23.6 | 5.7 | 35.3 | 0.8 |
| 08/jun/15 | Sf | N | -24.17 | -46.25 | 25 | -32 | 25 | 0 | 23.6 | 5.6 | 35.3 | 0.8 |
| 08/jun/15 | Mn | N | -23.95 | -45.90 | 1 | -23 | 8 | 0 | 23.9 | 6.2 | 35.2 | 0.8 |
| 08/jun/15 | Pb | N | -23.87 | -45.62 | 12 | -14 | 7 | 0 | 23.9 | 5.1 | 35.7 | 1.0 |
| 08/jun/15 | Sf | N | -24.05 | -45.62 | 8 | -5 | 1 | 2 | 23.3 | 5.7 | 35.8 | 1.0 |
| 09/jun/15 | Sg | N | -23.60 | -45.22 | 30 | -33 | 4 | 1 | 23.4 | 5.1 | 35.6 | 0.9 |
| 09/jun/15 | Dd | N | -23.47 | -44.75 | 120 | -25 | 10 | 0 | 23.8 | 6.2 | 35.3 | 0.8 |
| 09/jun/15 | Tt | N | -23.50 | -44.98 | 25 | -23 | 5 | 1 | 23.9 | 6.2 | 35.2 | 0.8 |

Species: Ba – *Balaenoptera ocutorostrata*; Bb – *Balaenoptera bonaerensis*; Be – *Balaenoptera edeni*; Dd – *Delphinus delphis*; Ea – *Eubalaena australis*; Mn – *Megaptera novaeangliae*; Oo – *Orcinus orca*; Pb – *Pontoporia blainvillei*; Sb – *Steno bredanensis*; Sf – *Stenella frontalis*; Sg – *Sotalia guianensis*; Tt – *Tursiops truncatus*.
